# How Nectar-Feeding Bats Localize their Food: Echolocation Behavior of *Leptonycteris yerbabuenae* Approaching Cactus Flowers

**DOI:** 10.1371/journal.pone.0163492

**Published:** 2016-09-29

**Authors:** Tania P. Gonzalez-Terrazas, Jens C. Koblitz, Theodore H. Fleming, Rodrigo A. Medellín, Elisabeth K. V. Kalko, Hans-Ulrich Schnitzler, Marco Tschapka

**Affiliations:** 1 Institute of Evolutionary Ecology and Conservation Genomics, University of Ulm, Ulm, Germany; 2 BioAcoustics Network, Neuss, Germany; 3 Department of Biology, University of Miami, Miami, Florida, United States of America; 4 Instituto de Ecología, Universidad Nacional Autónoma de México, México D. F., México; 5 Smithsonian Tropical Research Institute, Balboa, Panama; 6 Animal Physiology, Institute of Neurobiology, University of Tübingen, Tübingen, Germany; Università degli Studi di Napoli Federico II, ITALY

## Abstract

Nectar-feeding bats show morphological, physiological, and behavioral adaptations for feeding on nectar. How they find and localize flowers is still poorly understood. While scent cues alone allow no precise localization of a floral target, the spatial properties of flower echoes are very precise and could play a major role, particularly at close range. The aim of this study is to understand the role of echolocation for classification and localization of flowers. We compared the approach behavior of *Leptonycteris yerbabuenae* to flowers of a columnar cactus, *Pachycereus pringlei*, to that to an acrylic hollow hemisphere that is acoustically conspicuous to bats, but has different acoustic properties and, contrary to the cactus flower, present no scent. For recording the flight and echolocation behaviour we used two infrared video cameras under stroboscopic illumination synchronized with ultrasound recordings. During search flights all individuals identified both targets as a possible food source and initiated an approach flight; however, they visited only the cactus flower. In experiments with the acrylic hemisphere bats aborted the approach at ca. 40–50 cm. In the last instant before the flower visit the bats emitted a long terminal group of 10–20 calls. This is the first report of this behaviour for a nectar-feeding bat. Our findings suggest that *L*. *yerbabuenae* use echolocation for classification and localization of cactus flowers and that the echo-acoustic characteristics of the flower guide the bats directly to the flower opening.

## Introduction

The increasing interest in information acquisition in natural systems has resulted in the emergence of sensory ecology, the study of how organisms acquire and respond to information about their environment [1,2]. A striking example of such a sensory system is the echolocation of bats. From more than 1250 described bat species, approximately 1000 species use echolocation for spatial orientation and foraging [3].

The neotropical family Phyllostomidae with ca. 165 species has the highest ecological diversity among bats [4,5], with food resources used ranging from fruits, nectar and leaves, to insects, small vertebrates, and even blood [4,6]. The high ecological diversity of the phyllostomids is reflected neither in a high variability of echolocation call design nor in a large diversity of food acquisition strategies [7,8]. In general most phyllostomid species glean food items such as fruits, insects and small vertebrates from surfaces in highly cluttered environments [7,9–11]. Almost all phyllostomids recorded so far share a similar call structure: short (mostly ≤ 2 ms) frequency-modulated, multi-harmonic, broadband, and low-intensity signals. Consequently, they have been described as “whispering bats” [4,11–14]. Low intensity calls are seen as an adaptation to foraging in cluttered situations, as the use of loud echolocation calls within the vegetation could result in many loud echoes masking important information about the main targets [15]. However, recent studies have shown that at least some phyllostomid species emit echolocation calls at much higher intensities than expected and adjust the amplitude, i.e., the intensity of calls, over a large range when not foraging in gleaning mode [9,16,17].

Neotropical nectar-feeding bats (Glossophaginae: Phyllostomidae) spend much of their activity period on finding nectar-producing flowers. So far it is not well understood how bats find these and in particular, how they localize the flower opening. Most bat-pollinated plants produce a particular “musty” odor that aids bats in finding active flowers [18]. These volatile compounds present in the floral scents can be detected over long distances, hence scent is likely to be the primary cue for long-range detection of flowers, similar as in fruits [11,13,19]. Recent studies show that some bat-pollinated flowers have evolved characteristic acoustic reflective features that are conspicuous to bats and facilitate their detection by echolocation [20–22]. As echolocation may provide bats with precise information about flower position and floral structures, it is probable that echo-acoustic cues play a major role in flower classification and localization at short distances [20,21,23]. Vision might also play a role for some nectar-feeding bats, however mainly at crepuscular light or during moonlit nights and especially for finding foraging areas [24]. Because nectar-feeding bats, as well as fruit-eating bats, may combine scent (passive mode) and echolocation (active mode) for detecting and localizing their food they were assigned into a new guild called “narrow space passive/active gleaning bats” [23]. However, so far no study has focused on the echolocation behavior of nectar-feeding bats approaching open flowers. We don’t know if and to what extent these bats adjust their sonar system to the task on hand. The finding and localization of open flowers only by echolocation is a difficult task for most nectar-feeding bats because these often grow within dense vegetation and the bats have to discriminate between echoes from a potential food source and a multitude of background echoes. While most of the nectar-feeding bats forage in the extremely dense understorey of the tropical forest throughout the year, other species such as the migratory bat *Leptonycteris yerbabuenae* search, during part of the year, for flowering cacti in open deserts where the sensory task of finding active flowers by echolocation is more feasible. In this study we investigated the echolocation behavior of *L*. *yerbabuenae* approaching different targets. We also assessed whether these bats use echolocation for finding flowers and to what extent *L*. *yerbabuenae* adjusts its echolocation behavior in response to different targets. We conducted flight cage experiments comparing the flight and echolocation behavior of wild *L*. *yerbabuenae* when approaching two different targets: (1) the flower of a columnar cactus (*Pachycereus pringlei*) that has familiar acoustic and scent cues, and (2) a scentless acrylic hemisphere which is a completely unknown target for the bats. Previous studies showed that this object is acoustically conspicuous to nectar-feeding bats [25] but has different shape and texture to that of the natural flowers and these should translate into different acoustic characteristics. Using an artificial target which presents no scent and conspicuous acoustic cues will allow us to assess the role of echolocation for localization of potential food source. Besides, we can test if and to what extent these bats adjust their sonar system to the task on hand. We hypothesize that due to its ecology, i.e. migratory bat that during part of the year forages in habitats with very different vegetation structures and from very different types of flowers, *L*. *yerbabuenae* will present a plastic echolocation behavior and it would be able to detect, localize and visit both targets.

## Material and Methods

### Study animals and flight cage

*Leptonycteris yerbabuenae* is a relatively large (24–26 g) migratory New World nectar-feeding bat. This species mainly exploits pollen and nectar of tropical and subtropical plants that are adapted to bat pollination, especially columnar cacti (Cactaceae) and some species of *Agave* [26–30]. We captured *L*. *yerbabuenae* with mist nets placed in front of the entrance of a small cave near Kino Bay in the Mexican Sonoran Desert. The field work was conducted from mid-April to mid-May, which is the flowering peak of *Pachycereus pringlei*, one of the most common columnar cacti in the area [31].

All bats used in our experiments were wild individuals that had never been exposed to any artificial feeder and were used to forage at cactus flowers in the field. Behavioral experiments were conducted with 5 non-reproductive adult females in a flight cage that consisted of an aluminum frame (4m x 4m x 3m) covered with shade cloth of 70% light permeability. Prior to the behavioral experiments, bats were kept for one night in the flight cage for acclimation with *ad libitum* access to honey water at an artificial feeder that had no similarity with the targets later used in the experiments. The bats stayed in captivity between 2 and 4 days. The procedures performed in this study were not subject to approval of an institutional ethics committee as the home institution requires this only for domestic studies. Instead we followed the guidelines for the use of wild mammal species in research as recommended by the American Society of Mammalogists [32]. All captures were carried out under permission of the Secretaria de Medio Ambiente y Recursos Naturales, Mexico. This field permit (FAUT-0001) allowed us to capture and handle bats in the area of Bahia Kino, Mexico. All bats were released unharmed after the experiments at the capture site.

### Behavioral experiments

**Fig 1:**
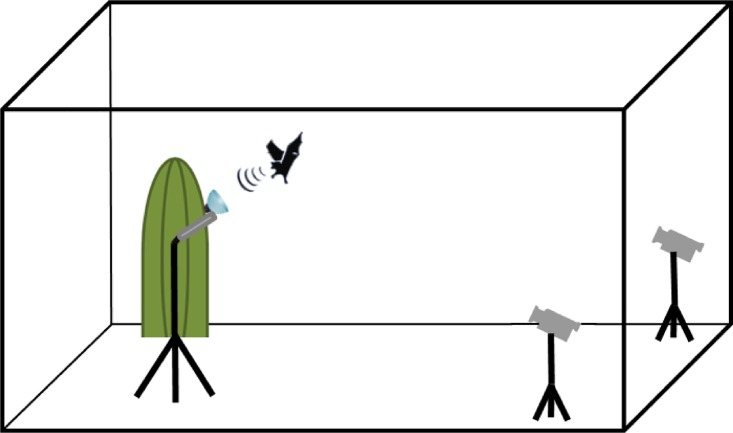
Experimental set up. Setup used during the flight cage experiments with both targets. We worked with one target at a time, respectively, each fixed to a cactus branch. The flight and echolocation behavior was recorded with an ultrasound microphone and two synchronized video cameras supported by stroboscopic light. Flight cage dimensions (4m x 4m x 3m).

During the behavioral experiments we exposed individuals of *L*. *yerbabuenae* to different objects (Fig 1A). As a natural target we used an open flower of the columnar cactus, *Pachycereus pringlei*, that is mainly pollinated by *L*. *yerbabuenae* [33–35], and as an artificial flower target we used an acrylic hollow hemisphere (10 cm diameter) that is acoustically conspicuous to bats [25]. Both targets were rewarded; every time a bat had visited a target we refilled it with ca. 100 **μ**l of sugar water (concentration 17%) to maintain a constant reward. All experiments were conducted at night in almost complete darkness to exclude visual cues. To present the experimental objects in the most natural way possible, we placed a straight upright branch of an adult *P*. *pringlei* (ca. 1.30 m) in one corner of the flight cage. On two ribs of the cactus we marked three different positions (6 in total) where we could fix the experimental targets. All targets were pointing slightly upward at an angle of ca. 30°; corresponding to the position of natural flowers. In the opposite corner of the flight cage we positioned two infra-red sensitive cameras (Sanyo IRP, Japan) to record the entire flight paths of a bat while approaching the targets (Fig 1B). These two cameras recorded 25 (interlaced) frames per second, each half-frame illuminated for 1 ms by a stroboscope with infra-red LEDs. Additionally we used a third camera (Sony Handycam, Japan) near the target to record details during the last part of the approach flight. To record the echolocation behaviour of *L*. *yerbabuenae* we used a custom-made AD-converting interface (PCTape, University of Tübingen, Germany) that allowed the synchronization of the sound and video recordings. Echolocation calls were recorded using a custom-made ultrasound microphone with a flat frequency response (±3dB between 18 kHz and 200 kHz) located on a tripod and oriented parallel to the target (Fig 1B). The recordings were digitalized with a sampling rate of 480 kHz and a resolution of 16 bit by the PCTape interface and stored as wav-files on a connected laptop.

Each individual was exposed to only one target at a time. The position where the target was presented to each bat was selected randomly. To avoid learning, consecutive targets were never placed in the same position. We recorded the first three approaches of each individual per target and scored an approach whenever a bat flew straight toward the target. A visit was counted when a bat inserted its snout into the target.

### Data analysis

#### Acoustic analysis

To analyze the sound recordings we used a custom-made software (Selena, University of Tübingen, Germany), using a Fast Fourier Transformation (FFT) analysis with a Hamming window and 512 samples, and a dynamic range of 60 dB (resolution after interpolation: 938 Hz / 0.066 ms). Start and end of the calls were defined as -15 dB below maximum amplitude. Statistics include mean ± SD unless stated otherwise. As *L*. *yerbabuenae* uses multi-harmonic calls, our analysis focused on the first harmonic, which consistently had the highest energy content. We measured pulse duration, pulse interval, initial and end frequency, bandwidth, and peak amplitude. As we did not record with a calibrated microphone, we have no information about the SPL of the echolocation calls; nevertheless it was possible to calculate the change in amplitude (dB) between consecutive calls of one flight path. Because we obtained the exact position of the bat while calling from the synchronization of the stereoscopic video and sound recording, we could estimate the expected loss in peak amplitude by distance (*ΔL*_*r*_) and by the directional sensitivity of the microphone (*ΔL*_*θ*_). By compensating for these losses, we obtained the total loss and subtracted it from the measured peak amplitude (A) in order to obtain the corrected peak amplitude emitted by the bat (*A*_*c*_):

$$A_{c}=A-\left(\Delta L_{r}+\Delta L_{\theta }\right),$$ (1)

To calculate the distance loss we used formula (2), where *r1* and *r2* are the distance of the bat to the target in two consecutive time instants [36].

$$\Delta L_{r}=20\;\log_{10}\;\frac{r2}{r1},$$ (2)

The additional decrease in signal amplitude caused by the directional sensitivity of the microphone was estimated, considering the worst case scenario, as a hypercardioid polar pattern [37], where θ1 and θ2 are the angles to the flower symmetry axis in two consecutive times:

$$\Delta L_{\theta }=20\;\log_{10}\;\left(\left|\frac{0.25+0.75\;\cos\theta_{2}}{0.25+0.75\;\cos\theta_{1}}\right|\right),$$ (3)

From these corrected peak amplitude values we determined whether changes in echolocation call volume during an approach were due just to physical factors or whether they were also generated by the bat. Because the distance was less that 1 m we did not take into account the atmospheric attenuation.

#### 3-D path reconstruction

We used the stereoscopic video recordings from the two infrared cameras to reconstruct three-dimensional flight paths of the bats and the position of the microphone and targets. The video analysis was done with the 3D Movement Analysis software (version 7.5.293, Simi Reality Motion Systems GmbH, Germany). In each video sequence the position of the head of the bat was marked on every half-frame (every 20 ms), and we calculated the flight path using a direct linear transformation. For the reconstruction of the flight path (reconstruction error ±5 cm) it was necessary to have the image of the bat in both cameras. Thus, the reconstruction began with the first frame where the bat was visible in both cameras, at ca. 1 m from the target and ended when the bat inserted its snout into the target or, in the case of an unsuccessful approach, when the bat turned away from the target. For a general characterization of the flight behavior we used the entire length of the video recordings.

The exact position of the bat at each call emission was interpolated from the position information available in 20 ms intervals. To compare the echolocation behavior of bats approaching the different targets, we grouped calls in ten distance intervals. In the plots the label assigned to each interval corresponds to the respective middle number: 0–<0.1 m, 0.05 m; 0.1 -<0.2 m, 0.15 m; 0.2–<0.3 m, 0.25 m; 0.3–<0.4 m, 0.35 m; 0.4–<0.5 m, 0.45 m; 0.5–<0.6 m, 0.55 m; 0.6–<0.7, 0.65 m; 0.7–<0.8, 0.75 m; 0.8–<0.9 m, 0.85 m; 0.9–<1 m, 0.95 m (range of the interval, middle value).

### Statistics

To test for significant differences in echolocation parameters (pulse duration, pulse interval, initial frequency, end frequency, and bandwidth) when approaching the two targets during the search phase, we used a Multivariate Analysis of Variance (MANOVA). To determine if distance interval, target type and the interaction between these parameters (distance*target) during the approach phase had an influence on call parameters we used a General Linear Mixed Model (GLMM) (normal distribution, identity link function). Individual bats were included as random effects. We also used a GLMM to test for statistical differences in call parameters among the last groups emitted while approaching both targets (terminal group, last group emitted before terminal group, last group emitted without visiting) and included the bat individual as a random effect. Sequential Bonferroni corrections were used for *post hoc* pairwise comparisons. Statistics were calculated using SPSS Statistics 17.0 (SPSS, IBM, USA).

## Results

### Flight behavior

**Fig 2:**
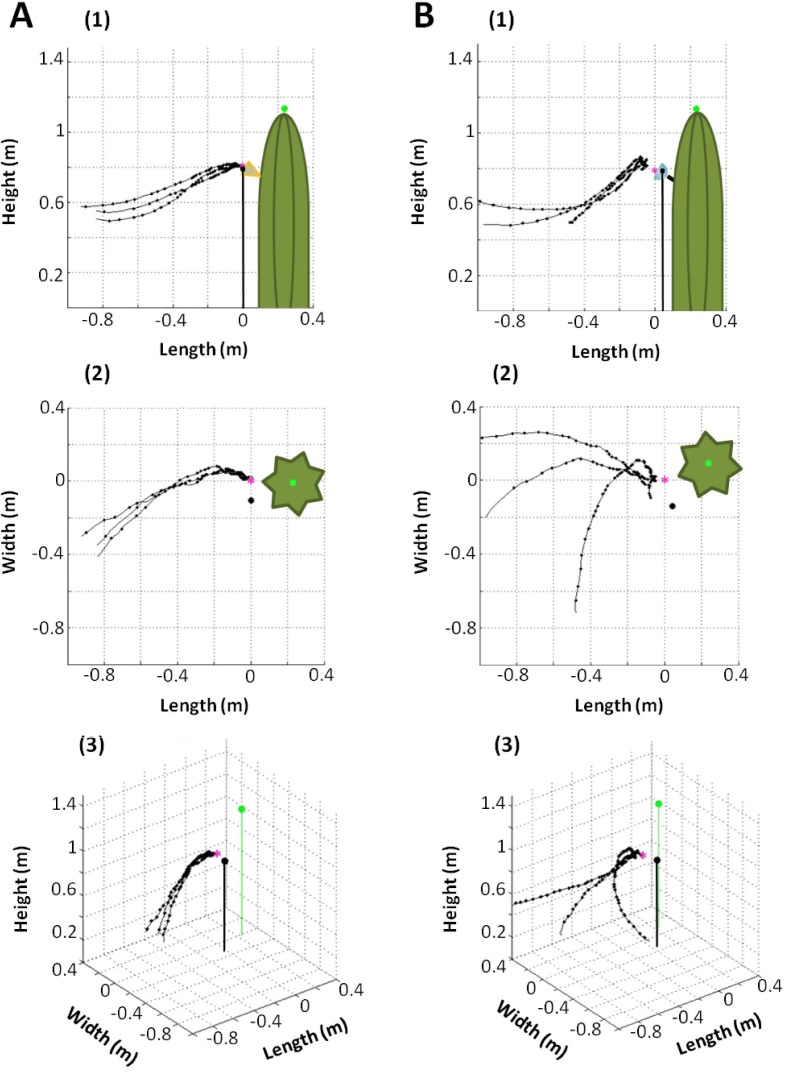
3D Flight paths reconstructions of a bat approaching the cactus flower and the hemisphere. Side (1), top (2) and 3D (3) view of three exemplary flight path reconstructions from one bat approaching cactus flower (A) and hemisphere (B). Small dots in the flight path show the position of the bat while emitting echolocation calls. Larger black and green dots represent the position of the microphone and the center of the cactus, respectively, and the pink star shows the position of the target.

We reconstructed a total of 15 sequences per target type (3 sequences per bat, n = 5 bats; see S1 Dataset). All individuals exhibited a very stereotyped behavior when visiting the cactus flower: before approaching the target, bats flew several times around the cactus at approximately 1–1.5 m above the ground (search flight). Before initiating an approach the bats decreased flight height, then they started the approach flight and flew up toward the open flower. At a distance of 95 cm the bats were 29±8 cm below the flower, and from this point on they flew up until they reached the flower (Fig 2A). Approximately 20 cm before reaching the flower, the bats were located 7.9±4.1 cm below the flower and always in front of the flower opening (Fig 2A). From there they proceeded to fly straight toward the flower until inserting their snout into it. All bats successfully visited the cactus flower and inserted almost the entire head into the flower’s corolla while hovering. During the experiments with the acrylic hemisphere, we did not observe the same stereotypic behavior as shown at the cactus flower. In general the flight trajectories were more variable, and bats approached from different sides and heights (Fig 2B). As with the cactus flower experiments, bats approached the target from below but, in contrast, at the end of the approach they usually rose above the target (Fig 2B). All individuals approached the hemisphere but never got closer than 6 cm. Bats hovered near the hemisphere, but after a few seconds they turned away. No individual attempted to insert its snout into the hemisphere.

### Echolocation behavior

**Fig 3:**
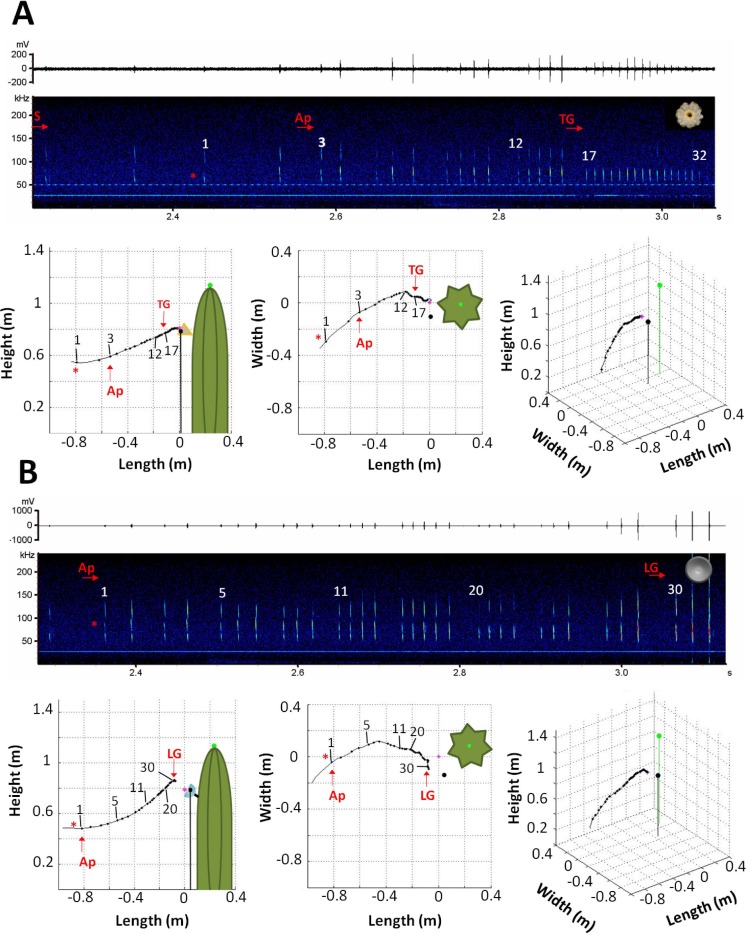
**Examples of flight and echolocation behavior (spectrograms) of a single bat approaching the cactus flower (A) and the hemisphere (B).** Small dots in the flight path show the position of the bat while emitting the echolocation calls. Larger black and green dots represent the position of the microphone and the center of the cactus, respectively, and the pink star shows the position of the target. The red asterisk indicates the first call that appears in the 3D flight path reconstruction. We could identify different phases: search (S), approach (Ap) and before inserting the snout into the cactus flower the bats broadcast an exceptionally long terminal group with increased call rate (TG). In contrast, when approaching the hemisphere, the last group (LG) emitted by the bats before giving up and flying away did not differ from other groups emitted during the entire approach. Spectrogram settings: FFT length: 512, window: Hamming and overlap: 50%.

**Fig 4:**
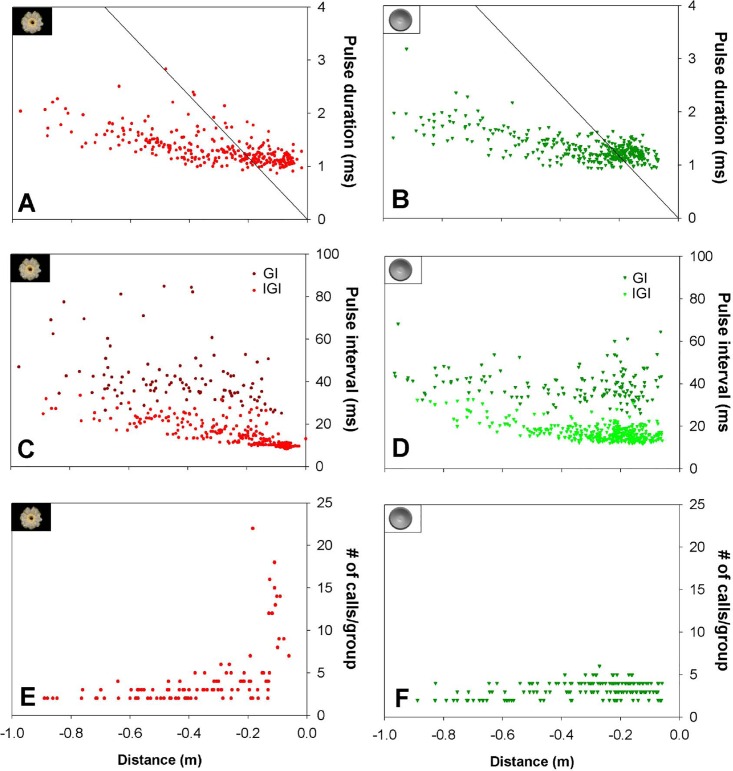
**Pulse duration (A and B), pulse interval (D and C) and number of calls per group (E and F) of all calls recorded from bats approaching cactus flower and hemisphere.** The line represents the beginning of the overlap zone and all calls located to the right of this line overlap with their echo. Pulse interval data are divided in inter-group interval (GI, higher values) and inter-pulse interval (IGI, smaller values). E and F represent the number of calls emitted per group while the bats approach to the targets; the plotted distance is the first call of each group.

All individuals were continuously echolocating throughout the search flight and the approach to both targets. During search flight (i.e., flight before the bats showed clear interest towards the target), bats emitted single, short, frequency-modulated (FM) and multi-harmonic echolocation calls. We found no significant difference in any call parameter between targets during search (F_4, 5_ = 0.20, P < 0.925; Wilk's λ = 0.859; Table 1). As the search calls were in general emitted at a distance of more than 1 m to the targets (outside the view of both cameras), we had no information about the position of the bats while they were emitting these calls. Upon closing in on the target, bats changed to typical approach calls, characterized by the emission of call groups with an increasing number of calls of decreasing pulse duration and pulse interval (Figs 3 and 4). We observed the main difference between approaches to the different targets in the number of calls in the last group of calls emitted before the bat visited the target or decided to fly away. Just before bats inserted their snout into the cactus flower, they emitted a long group with an average of 13±4 calls, which we define as “long terminal group” (Fig 3A). In contrast, none of the individuals made the attempt to visit the hemisphere, and the last call group emitted before turning away always consisted of only 2–4 calls; we will refer to this as “last group” (Fig 3B).

**Table 1:** Comparison of call parameters (mean ± SD) during search phases at both targets. We found no significant difference in call parameters between targets p > 0.05.

**Search phase**						
**Call parameters**	**Flower**			**Hemisphere**		
	n= 60			n= 60		
Pulse interval (ms)	90.0	±	10.6	90.3	±	5.9
Pulse duration (ms)	2.2	±	0.6	2.57	±	1.1
Initial frequency (kHz)	85.3	±	0.9	84.9	±	3.6
End frequency (kHz)	52.4	±	4.3	50.9	±	4.0
Bandwidth (kHz)	33.0	±	3.9		34	±	3.3

### Approach call parameters

**Fig 5:**
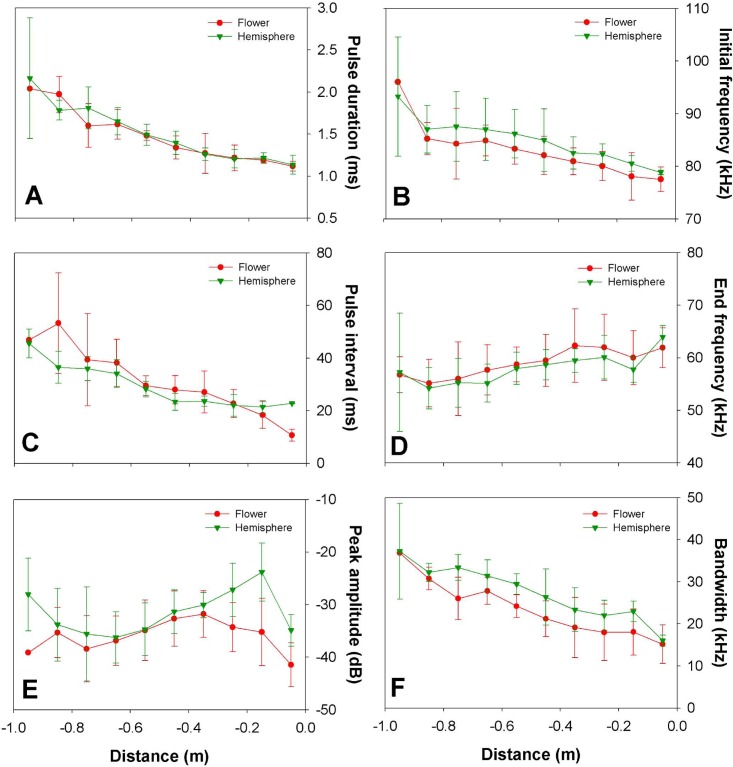
Echolocation call parameters during the approach to the cactus flower and the hemisphere. Pulse duration (A), initial frequency (B), pulse interval (C), end frequency (D), peak amplitude (E) and bandwidth (F) per distance interval (mean values ± SD) during the approach to the flower and hemisphere targets. The peak amplitude was corrected for the expected decrease of 6 dB per halving of the distance and by angle flight (θ, φ) with respect to the target.

We observed characteristic changes in the call parameters between targets and depending on distance during the approach phase. We only found a significant effect of distance on the pulse duration (PD), while target type and their interaction showed no significant effects (GLMM; distance: F_9, 62.68_ = 15.28, p<0.0001; target: F_1, 62.72_ = 0.347, p = 0.558; distance*target: F_9, 62.41_ = 0.431, p = 0.913; AIC = 11.621). For both targets pulse duration decreased as bats approached the target; the closer the bat came to the target, the shorter the calls became (Figs 4A, 4B and 5A). Pulse duration decreased by 55% between the farthest and the closest distance interval (farthest: 2.04±0.21 ms, closest: 1.12±0.058 ms). Using pulse duration and the exact position of the bat while calling, we could determine the calls where the bat experienced overlap between emitted call and returning echo. For calculation of sound speed we took into account the atmospheric conditions such as relative humidity, temperature and air pressure. In general, calls of the last part of the approach overlapped with returning echoes, e.g., from a distance of 15 cm to the target, 96% of the calls overlapped with the returning echo (Fig 4A and 4B).

Only distance had a significant effect on pulse interval (PI) (GLMM; distance: F_9, 68.11_ = 18.953, p<0.0001; target: F_1, 68.10_ = 2.237, p = 0.139; distance*target: F_9, 68.12_ = 2.428, p<0.019; AIC = 512.105). At both targets PI decreased with decreasing distance to the target during the first stage of the approach (1m-50cm; Fig 5C). Nevertheless, at the cactus flower the PI decreased constantly during the entire approach whereas in approaches to the hemisphere the PI stopped decreasing at a distance of 45 cm from the target (Fig 5C). At a distance of ca. 12 cm from the flower all emitted calls were part of the terminal group and had the shortest mean pulse interval of 9.8±0.08 ms (Table 2). Bats stopped echolocating when they were 3.7±1.2 cm away from the flower (Table 2) and then inserted their snout into the flower opening. During the approaches to the hemisphere, none of the bats approached closer than 6 cm to the artificial target. Bats started broadcasting the “short last group” before turning away at an average of 17.5±6.9 cm from the hemisphere (Table 2). During approaches to both targets, bats broadcast groups of calls that generally ranged from 2–5 calls per group. The maximum number of calls per group was always found in the “long terminal group” broadcast immediately before the visit to a flower. The bats emitted 60% more groups when approaching the hemisphere but almost never emitted groups with more than 5 calls (flower: 103 groups, hemisphere: 170 groups). Plotting of PI against distance results in two separate clouds (Fig 4C and 4D) that represent the intra-group pulse interval (IGI, smaller values) and the inter-group interval (GI, higher values). During approaches to the cactus flower, bats started to increase the number of calls per group at a distance of ca. 40 cm from the flower (Fig 4E). However, when approaching the hemisphere bats emitted mainly groups of 3–4 calls until the end of the approach but increased the number of groups (Fig 4F).

**Table 2:** Comparison of the last groups of calls emitted: immediately before visiting the cactus flower (terminal group) or before emitting the terminal group (last group before TG), or at the hemisphere just before giving up and flying away (last group).

	**Cactus Flower**							** **	**Hemisphere**				
% visits	87%								0%				
	**Terminal group**				**Last group before TG**				**Last group**				
# of calls	12	±	4	a	4	±	1	b	3	±	0.4	b	**
Distance bat – target at first call (cm)	10.8	±	2.0	a	19	±	2	b	16.4	±	6.8	ab	*
Distance bat – target at last call (cm)	3.7	±	1.2	a	14.7	±	1.9	b	17.5	±	6.9	b	**
Speed (m/s)	0.9	±	0.2	a	1.2	±	0.3	b	0.9	±	0.2	a	**
Pulse interval (ms)	9.8	±	0.8	a	15.0	±	3.1	b	19.8	±	4.6	b	**
Pulse duration (ms)	0.8	±	0.1	a	1.0	±	0.21	ab	1.1	±	0.2	b	**
Initial frq. (kHz)	81.2	±	1.5	a	81.9	±	3.8	a	86.3	±	2.6	a	
Final frq. (kHz)	57.2	±	3.8	a	54.7	±	5.0	a	49.7	±	4.5	b	**
% calls with overlap	98%			a	60%			a	60%			a	

Values are means ± SD. Different letters within a row indicate significant differences

*p<0.01,

** p<0.001

We also observed effects of distance and target type on frequency parameters. Distance interval had a significant effect on the initial frequency (IF) (GLMM; distance: F_9, 68.08_ = 7.272, p<0.0001; target: F_1, 68.07_ = 3.938, p = 0.051; distance*target: F_9, 68.08_ = 0.163, p = 0.997; AIC = 444.608). At both targets bats lowered the IF when getting closer to the target (Fig 5B). During the entire approach bats tended to consistently emit higher IF when approaching the hemisphere than the cactus flower (Fig 5B). There was no significant difference in end frequency (EF) at different targets, but we observed a trend to emit lower EF when approaching the hemisphere. Distance to the target had an effect on the EF, but, contrary to the IF, the end frequency increased when bats got closer to the target (GLMM; distance: F_9, 365.13_ = 3.205, p = 0.003; target: F_1, 1.20_ = 1.196, p = 0.278; distance*target: F_9, 65.10_ = 0.302, p = 0.971; AIC = 430.981) (Fig 5D). The tendencies towards higher IF and lower EF resulted in a significantly higher bandwidth during the approach to the hemisphere (Fig 5F). Distance also had a significant effect on bandwidth, which decreased significantly when bats got closer to both target types (GLMM; distance: F_9, 63.45_ = 9.908, p<0.0001; target: F_1, 63.60_ = 9.901, p = 0.003; distance*target: F_9, 63.06_ = 0.301, p = 0972; AIC = 433.017).

The distance to the target, target type and their interaction had also an effect on the peak amplitude of the calls (GLMM; distance: F_9, 65.04_ = 4.183, p<0.0001; target: F_1, 65.15_ = 25.793, p<0.0001; distance*target: F_9, 65.04_ = 2.844, p<0.0001; AIC = 425.716). At the initial part of the approach the peak amplitude was similar at both targets. However, during approaches to the cactus flower, peak amplitude started to diminish at ca. 40 cm from the flower and continued decreasing during the remaining approach phase (Fig 5E). In contrast, during the approach to the hemisphere, peak amplitude did not decrease but continued to increase (Fig 5E). In general, at close distances bats emitted louder calls when approaching the hemisphere than when approaching the cactus flower.

## Discussion

At both targets bats started approach flight at a distance of ca. 1 m– 80 cm. This suggests that both targets provided at this position echoes that could be discriminated from the echoes of the cactus stem. We assume that loud echoes, such as reported for the hemisphere and some bat-pollinated flowers [20,22,25], indicated to the bat the presence of a possible food source (open flower) and triggered the approach. Nevertheless, *Leptonycteris yerbabuenae* only visited the cactus flower and in contrast to our initial hypothesis no individual made an attempt to insert its snout into the hemisphere. Our comparison of the flight and echolocation behavior of *L*. *yerbabuenae* when approaching a cactus flower vs. an artificial hemisphere revealed distinct and target-type-dependent differences in bat behavior and call parameters.

### Approach flight behavior

All bats showed a rather stereotyped flight behavior while approaching the cactus flower: after detection they approached the target from below until reaching the flower opening, then they inserted the snout for nectar extraction while hovering. A similar approach behavior from below has been reported for other fruit- and nectar-feeding phyllostomid bats [9,19,38]. At a distance of ca. 20 cm the bats were approximately 8 cm below the flower opening and still flying upward in a straight line. In contrast, approach behavior was distinctly different when bats were flying toward the hemisphere target. Approach flight paths were not so stereotyped and started at different parts of the flight cage. Sometimes the flight direction was changed during the approach, and bats never got closer than 6 cm to the target. At a distance of 40–30 cm away, bats started to fly above the hemisphere before turning away. These differences in approach behavior indicate that the bats discriminated the two targets at a distance of 40–30 cm.

The spectral composition of the echoes of objects may be an important character for target discrimination [22,39]. In bell-shaped flowers the spectral composition of echoes depends strongly on the angle of incidence, and the echo is always strongest when the echolocating bat is positioned on axis with the opening (0°) [22]. Hence, a bat flying toward a bell-shaped flower can find the flower opening by evaluating changes in strength and spectral composition of the returning echoes [22]. In contrast, the spectral composition of echoes reflected by a hemisphere is rather independent of the angle of incidence, and the maximum in echo amplitude at 0° is less pronounced [25,39]. Bats obtain this information not from a single call, but from the analysis of sequential calls during the approach flight that successively generates an “acoustical image” of the target [40,41]. The latter may explain why bats showed such stereotyped flight behavior during the approach to cactus flowers: they approached every time approximately from the same angle and therefore they obtained similar acoustic information during an approach. In contrast to those from bell-shaped flowers, power spectra of a hemisphere do not change as distinctly with the angle of incidence [25]. The more erratic flight behavior of bats during the approach to the hemisphere may reflect the effort of the bats to pinpoint changes in the echo patterns that could provide them with information about the location of the ‘flower’ opening. All the bats that we used in the experiments were wild bats caught during the flowering season of *P*. *pringlei*, which means that these bats already knew and most probably had visited a cactus flower before. In contrast, the hemisphere is a completely unknown target for the bats. Therefore, none of them associated this target with a potential food source during the brief time of the experiment and never visited the hemisphere. Probably the more homogenous reflections of this simulated flower target generated an “acoustic image” that provided less directional information and completely different from the “acoustic image” of the familiar natural flowers.

### Echolocation behavior during approach flights

The general structure of calls emitted while approaching both targets was similar: short, multi-harmonic and broadband FM calls. This call type is well suited for precise localization of a target and for separating a target from the background [15,41,42]. However, as we hypothesized, we found significant differences in the calling pattern and in some call parameters that suggest that *L*. *yerbabuenae* adjusts its echolocation behavior according to the information it receives from the target of interest. We found that independent from target type bats modified the duration of their calls, depending on the distance to the target. The duration of the calls decreased with decreasing target distance, similar to other FM bats, probably to avoid a pulse-echo overlap [19,43]. Through the reduction of call duration *L*. *yerbabuenae* can avoid overlap at distances greater than 20 cm, but when closer than this, 96% of the echoes overlap with the calls (Fig 4B). The bats may be able to deal with this because FM calls are less overlap-sensitive than longer, narrowband calls used by many open space aerial insectivorous bat species [44].

During approaches to the cactus flower, the pulse interval was continuously reduced up to the end of the approach, whereas at approaches to the hemisphere bats stopped the pulse interval reduction after realizing that the target was not a flower. In this case, animals did not continue the approach and started to turn away. Pulse interval at the hemisphere did not shorten any more at distances below 50 cm, while it continued to decrease at the cactus flower. This suggests that at this distance bats had recognized the difference between the targets and aborted the approach to the hemisphere. This difference is especially evident in the long terminal group, characterized by very short pulse intervals between calls, which was emitted only at the end of visits to the cactus flower.

Also, bats broadcast a higher number of call groups while approaching the hemisphere, mainly during the last part of the approach. Grouping of calls has been reported for several phyllostomids and is also typical in FM bat species such as *Eptesicus fuscus* [9,19,41,45–47]. An increase in the number of call groups occurs when bats are exposed to acoustically difficult situations and might help them to obtain a better spatial representation of targets and background [41,46,47]. After aborting the approach, bats were already very near to the cactus that was located in one of the corners of the flight cage. The increased number of groups suggests that the bats were trying to orient themselves in this cluttered situation while changing the flight direction and flying away.

Regarding call amplitude, bats behaved similarly during the first part of the approach to both targets. However, starting at a distance of ca. 40 cm from the cactus flower, the peak amplitude started to decrease. This corresponds to recent studies showing that bats generally reduce call intensity, when getting closer to the target [9,48,49]. In approaches to the hemisphere, at a distance of ca. 40–50 cm bats apparently realized this target was not a flower, aborted the approach, and increased the peak amplitude of their calls, indicating that their flight and echolocation behavior was no longer directed at and controlled by the hemisphere target.

When approaching the hemisphere, bats tended to emit higher bandwidth FM calls. Such signals support precise localization and may also be used for target discrimination and classification (e.g., size, shape, texture) [15,50]. The higher bandwidth at the beginning of the approach (100–50 cm) may have helped during the initial target discrimination. Admittedly, the encountered differences in frequency parameters are relatively small, so we cannot be sure that they actually reflect deliberate behavioral adjustments. Changes in temporal and intensity patterns of the calls indicate that at a distance of ca. 50 cm the bats acoustically discriminated between the targets. Differences in bandwidth between the targets during the last stage of the approach (50 cm onwards) therefore probably mainly reflect different behavior as bats continued to approach the flower but turned away from the hemisphere.

In summary, we observed that bats exhibited similar behavior at the two target conditions not only during the search phase but also at the beginning of the approach, at a distance between ca. 1 m and 50 cm from the target. This suggests that the echoes reflected from a cactus branch with a hemisphere target and from a cactus branch with an open flower initially indicated the potential presence of food. However, at ca. 50 cm from the hemisphere, bats sensed the difference between the echoes and realized that the object in front of them was not a cactus flower. Therefore, after this point they did not continue to develop the echolocation behavior into a typical final approach phase but aborted the approach.

### Terminal group

Before inserting their snout into the cactus flower all individuals of *L*. *yerbabuenae* broadcast a long terminal group of 10–20 calls of short duration (0.8 ± 0.1 ms) and short pulse interval (9.8 ± 0.8 ms). This is the first report of this behavior for a nectar-feeding bat. Until now only one phyllostomid species was known to exhibit this kind of terminal-buzz-like phase, the insectivorous trawling bat *Macrophyllum macrophyllum* [9,51]. The absence of a typical terminal buzz in phyllostomids has usually been explained by their main sensory requirements during foraging: as almost all members of this family feed on motionless objects, such as fruits or insects that are in dense vegetation, there is no need for an increase in information flow, compared to when feeding on fast moving insects [13,19,51]. The fact that the flower-visiting *Leptonycteris* emits a long terminal group immediately before visiting a flower confirms our hypothesis that this species uses echolocation for locating a flower and, in particular, the flower opening. By emitting this terminal group bats obtain exact information not only about the location of the flower, but also on flower structures (e.g., the corolla) that can be a good guide to the nectar resources. This behavior allows these bats to visit the flower almost without hesitation and error, which may play a particularly important role when visiting plants with long spines, such as columnar cacti, where even small navigational errors may turn out to be fatal.

The echoes reflected from the inside of the long tube of the cactus flower might function as an acoustic guide that provides the bat with detailed information on the location of the flower opening and the orientation of the floral tube [22]. In addition, cacti as well as many bat-pollinated flowers have particularly robust and rigid petals [22] that may indicate the flower opening not only visually but also acoustically. *Leptonycteris yerbabuenae* only emitted the long terminal group while approaching the flower. We suggest that specific echo-acoustic characteristics of the flower guide the bats directly into the opening of the flower, and emission of an extended terminal group of calls at the end of the approach sequence aids this task.

Like many other bats, fruit- and nectar-feeding species use echolocation for orientation in space and obstacle avoidance. For finding food these bats probably use a combination of different sensory systems sequentially; odor and vision for a more diffuse long range detection of resources, and echolocation during the actual short range approach that permits a precise localization of the flower or fruit [11,18,19,23,42,50]. Our data suggest that at short distance nectar-feeding bats use echolocation for locating the presence of potential food and particularly for finding the exact position of the flower opening. Nevertheless, because the bats never visited the scentless hemisphere we cannot fully exclude the supplementary use of scent for target recognition at short range.

In conclusion, our evidence suggests that *Leptonycteris yerbabuenae* rely heavily on echolocation for detection and localization of the flower, and more specifically of the flower opening. The significance of a terminal buzz for capturing prey is well documented for aerial insectivores foraging in open or edge space [23,42,50,52,53]. So far, the trawling *Macrophyllum macrophyllum* and now also *L*. *yerbabuenae* are the only phyllostomid species that also broadcast a long group of echolocation calls just before feeding. Both of these species forage in more open areas, which suggests that species of this family might have a greater flexibility in echolocation behavior than currently believed, influenced by ecological factors, such as foraging behavior and habitat use [9,54].

## Supporting Information

S1 Dataset 3D flight path reconstruction data. (XLSX)

## References

[1] Chittka L, Thomson JD, editors. Cognitive Ecology of Pollination: Animal Behaviour and Floral Evolution Cambridge University Press; 2001.

[2] Dangles O, Irschick D, Chittka L, Casas J. Variability in sensory ecology: expanding the bridge between physiology and evolutionary biology. Quarterly Review of Biology. 2009;84: 51–74. 10.1086/596463 19326788

[3] Simmons NB, Wetterer AL. Estimating diversity: how many bat species are there? 15th International Bat research Conference. Praha; 2009.

[4] Kalko EK V. Neotropical leaf-nosed bats (Phyllostomidae): “Whispering” bats or candidates for acoustic survey? In: Brigham M, Jones G, Kalko EK V, editors. Proceedings of a workshop on identification and acoustic monitoring of bats. Austin, Texas: Bat Conservation International; 2002. pp. 63–69.

[5] Simmons NB. Order Chiroptera In: Wilson DE, Reeder DM, editors. Mammal species of the World: a taxonomic and geographic reference. Johns Hopkins University Press; 2005 pp. 312–529.

[6] Findley JS. Bats: a community perspective 1st ed. New York, USA: Cambridge Univ. Press; 1993.

[7] Kalko EK V, Handley COJ, Handley D. Organization, diversity and long-term dynamics of a neotropical bat community In: Cody M, Smallwood J, editors. Long-term studies in vertebrate communities. Los Angeles: Academic Press; 1996 pp. 503–553.

[8] Kalko EK V, Herre EA, Handley COJ. Relation of fig fruit characteristics to fruit-eating bats in the New and Old World tropics. J Biogeogr. 1996;23: 565–576. 10.1111/j.1365-2699.1996.tb00018.x

[9] Brinkløv S, Kalko EK V, Surlykke A. Intense echolocation calls from two “whispering” bats, Artibeus jamaicensis and Macrophyllum macrophyllum (Phyllostomidae). The Journal of experimental biology. 2009;212: 11–20. 10.1242/jeb.023226 19088206

[10] Geipel I, Jung K, Kalko EK V. Perception of silent and motionless prey on vegetation by echolocation in the gleaning bat Micronycteris microtis. Proceedings of the Royal Society B: Biological Sciences. 2013;280: 20122830 10.1098/rspb.2012.2830 23325775 PMC3574334

[11] Korine C, Kalko EK V. Fruit detection and discrimination by small fruit-eating bats (Phyllostomidae): echolocation call design and olfaction. Behav Ecol Sociobiol. 2005;59: 12–23. 10.1007/s00265-005-0003-1

[12] Griffin DR, Novick A. Acoustic orientation of neotropical bats. Journal of Experimental Zoology. 1955;130: 251–300. 10.1002/jez.1401300205

[13] Kalko EK V, Condon MA. Echolocation, olfaction and fruit display: how bats find fruit of flagellichorous cucurbits. Funct Ecol. 1998;12: 364–372. 10.1046/j.1365-2435.1998.00198.x

[14] Weinbeer M, Meyer CF, Kalko EK V. Activity pattern of the trawling phyllostomid bat, Macrophyllum macrophyllum, in Panama. Biotropica. 2006;38: 69–76.

[15] Schnitzler HU, Kalko EK V. Echolocation by insect-eating bats. BioScience. 2001;51: 557–569. 10.1641/0006-3568(2001)051

[16] Brinkløv S, Jakobsen L, Ratcliffe JM, Kalko EK V, Surlykke A. Echolocation call intensity and directionality in flying short-tailed fruit bats, Carollia perspicillata (Phyllostomidae). Journal of the Acoustical Society of America. 2011;129: 427–435. 10.1121/1.3519396 21303022

[17] Mora EC, Macías S. Echolocation calls of Poey’s flower bat (Phyllonycteris poeyi) unlike those of other phyllostomids. Die Naturwissenschaften. 2007;94: 380–383. 10.1007/s00114-006-0198-7 17149582

[18] von Helversen O, Winkler L, Bestmann HJ. Sulphur-containing “perfumes” attract flower-visiting bats. J Comp Physiol A Sens Neural Behav Physiol. 2000;186: 143–153. 10.1007/s003590050014 10707312

[19] Thies W, Kalko EK V, Schnitzler HU. The roles of echolocation and olfaction in two Neotropical fruit-eating bats, Carollia perpiscillata and C. castanea, feeding on Piper. Behav Ecol Sociobiol. 1998;42: 397–409. 10.1007/s002650050454

[20] Simon R, Holderied MW, Koch CU, von Helversen O. Floral acoustics: conspicuous echoes of a dish-shaped leaf attract bat pollinators. Science. 2011;333: 631–633. 10.1126/science.1204210 21798950

[21] von Helversen D, von Helversen O. Object recognition by echolocation: a nectar-feeding bat exploiting the flowers of a rain forest vine. Journal of comparative physiology A, Neuroethology, sensory, neural, and behavioral physiology. 2003;189: 327–36. 10.1007/s00359-003-0405-3 12712362

[22] von Helversen D, Holderied MW, von Helversen O. Echoes of bat-pollinated bell-shaped flowers: conspicuous for nectar-feeding bats? J Exp Biol. 2003;206: 1025–1034. 10.1242/jeb.00203 12582145

[23] Denzinger A, Schnitzler H-U. Bat guilds, a concept to classify the highly diverse foraging and echolocation behaviors of microchiropteran bats. Frontiers in physiology. Frontiers; 2013;4: 164 10.3389/fphys.2013.00164 23840190 PMC3699716

[24] Rydell J, Eklöf J. Vision complements echolocation in an aerial-hawking bat. Die Naturwissenschaften. 2003;90: 481–3. 10.1007/s00114-003-0464-x 14564410

[25] Simon R, Holderied MW, von Helversen O. Size discrimination of hollow hemispheres by echolocation in a nectar feeding bat. J Exp Biol. 2006;209: 3599–3609. 10.1242/jeb.02398 16943500

[26] Arita HT. Spatial segregation in long-nosed bats Leptonycteris nivalis and Leptonycteris curasoae in Mexico. J Mammalogy. 1991;72: 706–714. 10.2307/1381831

[27] Gardner AL. Feeding habits Baker RJ, Carter DC, editors. Special publications the Museum Texas Tech University. Special Pu. Mus. Texas Tech Univ; 1977;13: 293–350.

[28] Moreno-Valdez A, Honeycutt RL, Grant WE. Colony dynamics of Leptonycteris nivalis (Mexican long-nosed bat) related to flowering agave in Northern Mexico. J Mammalogy. 2004;85: 453–459. 10.1644/1545-1542(2004)085<0453:CDOLNM>2.0.CO;2

[29] Sánchez R, Medellín RA. Food habits of the threatened bat Leptonycteris nivalis (Chiroptera: Phyllostomidae) in a mating roost in Mexico. Journal of Natural History. 2007;41: 1753–1764. 10.1080/00222930701483398

[30] Valiente-Banuet A, Arizmendi M d. C, Rojas-Martinez A, Dominguez-Canseco L. Ecological relationships between columnar cacti and nectar-feeding bats in Mexico. Journal of Tropical Ecology. 1996;12: 103–119. 10.1017/S0266467400009330

[31] Fleming TH, Tuttle MD, Horner MA. Pollination Biology and the Relative Importance of Nocturnal and Diurnal Pollinators in Three Species of Sonoran Desert Columnar Cacti. The Southwestern Naturalist. 1996;41: 257–269.

[32] Sikes RS, Gannon WL. Guidelines of the American Society of Mammalogists for the use of wild mammals in research. Journal of Mammalogy. American Society of Mammalogists Illinois Natural History Survey, 1816 South Oak Street, Champaign, IL 61820; 2011;92: 235–253. 10.1644/10-MAMM-F-355.1

[33] Fleming T, Maurice S, Hamrick JL. Geographic variation in the breeding system and the evolutionary stability of trioecy in Pachycereus pringlei (Cactaceae). Evolutionary Ecology. Springer Netherlands; 1998;12: 279–289. 10.1023/a:1006548132606

[34] Fleming TH, Sahley CT, Holland JN, Nason JD, Hamrick JL. Sonoran Desert columnar cacti and the evolution of generalized pollination systems. Ecological Monographs. 2001;71: 511–530. 10.2307/3100034

[35] Molina-Freaner F, Eguiarte L. The pollination biology of two paniculate Agaves (Agavaceae) from northwestern Mexico: contrasting roles of bats as pollinators. American Journal of Botany. 2003;90: 1016–1024. 10.3732/ajb.90.7.1016 21659200

[36] Kinsler LE, Frey AR, Coppens AB, Sanders J V. Fundamentals of Acoustics. 4th ed. New York, USA: John Wiley & Sons, Ltd; 2000.

[37] Eargle J. Handbook of recording engineering 4th ed. Norwel, Massachusetts: Kluwer Academ Publishers; 2003.

[38] Gonzalez-Terrazas TP, Medellin RA, Knörnschild M, Tschapka M. Morphological specialization influences nectar extraction efficiency of sympatric nectar-feeding bats. The Journal of experimental biology. 2012;215: 3989–3996. 10.1242/jeb.068494 22899529

[39] von Helversen D. Object classification by echolocation in nectar feeding bats: size-independent generalization of shape. Journal of comparative physiology A, Neuroethology, sensory, neural, and behavioral physiology. 2004;190: 515–21. 10.1007/s00359-004-0492-9 15103497

[40] Moss CF, Surlykke A. Auditory scene analysis by echolocation in bats. Journal of the Acoustical Society of America. 2001;1: 2207–2226. 10.1121/1.1398051 11681397

[41] Moss CF, Bohn K, Gilkenson H, Surlykke A. Active listening for spatial orientation in a complex auditory scene. Plos Biology. 2006;4: 615–626. 10.1371/journal.pbio.0040079 PMC1393756 16509770

[42] Kalko EK V, Schnitzler HU. How echolocating bats approach and acquire food In: Kunz TH, Racey PA, editors. Bat biology and conservation. Washington: Smithsonian Institution Press; 1998 pp. 197–204.

[43] Surlykke A, Pedersen SB, Jakobsen L. Echolocating bats emit a highly directional sonar sound beam in the field. Proceedings of the Royal Society Biological Sciences Series B. 2009;276: 853–860. 10.1098/rspb.2008.1505 19129126 PMC2664374

[44] Schnitzler HU, Kalko EK V. How echolocating bats search for and find food. In: Kunz TH, Racey PA, editors. Bat biology and conservation. 1998. pp. 183–196.

[45] Petrites AE, Eng OS, Mowlds DS, Simmons JA, DeLong CM. Interpulse interval modulation by echolocating big brown bats (Eptesicus fuscus) in different densities of obstacle clutter. Journal of comparative physiology A, Neuroethology, sensory, neural, and behavioral physiology. 2009;195: 603–17. 10.1007/s00359-009-0435-6 19322570

[46] Sändig S, Schnitzler H-U, Denzinger A. Echolocation behaviour of the big brown bat (Eptesicus fuscus) in an obstacle avoidance task of increasing difficulty. The Journal of experimental biology. 2014;217: 2876–84. 10.1242/jeb.099614 24902745

[47] Kothari NB, Wohlgemuth MJ, Hulgard K, Surlykke A, Moss CF. Timing matters: sonar call groups facilitate target localization in bats. Frontiers in physiology. 2014;5: 168 10.3389/fphys.2014.00168 24860509 PMC4026696

[48] Surlykke A, Kalko EK V. Echolocating bats cry out loud to detect their prey. PLoS ONE. 2008;3: e2036 10.1371/journal.pone.0002036 18446226 PMC2323577

[49] Koblitz JC, Stilz P, Pflästerer W, Melcón ML, Schnitzler H-U. Source level reduction and sonar beam aiming in landing big brown bats (Eptesicus fuscus). The Journal of the Acoustical Society of America. Acoustical Society of America; 2011;130: 3090–9. 10.1121/1.3628345 22087937

[50] Schnitzler HU, Moss C, Denzinger A. From spatial orientation to food acquisition in echolocating bats. Trends in ecology and evolution. 2003;18: 386–394. 10.1016/S0169-5347(03)00185-X

[51] Weinbeer M, Kalko EK V. Ecological niche and phylogeny: the highly complex echolocation behavior of the trawling long-legged bat, Macrophyllum macrophyllum. Behavioral Ecology and Sociobiology. 2007;61: 1337–1348. 10.1007/s00265-007-0364-8

[52] Moss CF, Surlykke A. Probing the natural scene by echolocation in bats. Frontiers in behavioral neuroscience. 2010;4: 1–16. 10.3389/fnbeh.2010.00033 20740076 PMC2927269

[53] Surlykke A, Futtrup V, Tougaard J. Prey-capture success revealed by echolocation signals in pipistrelle bats (Pipistrellus pygmaeus). J Exp Biol. 2003;206: 93–104. 10.1242/jeb.00049 12456700

[54] Weinbeer M, Kalko EK V., Jung K. Behavioral flexibility of the trawling long-legged bat, Macrophyllum macrophyllum (Phyllostomidae). Frontiers in Physiology. Frontiers; 2013;4 10.3389/fphys.2013.00342 24324442 PMC3838978

